# Association among Disorders of Gut-Brain Interaction (DGBI) and Fibromyalgia: A Prospective Study

**DOI:** 10.3390/jcm11030809

**Published:** 2022-02-03

**Authors:** Carmela Settembre, Elvira D’Antonio, Paolo Moscato, Gabriella Loi, Antonella Santonicola, Paola Iovino

**Affiliations:** 1 Gastrointestinal Unit Department of Medicine, Surgery and Dentistry “Scuola Medica Salernitana”, University of Salerno, 84081 Baronissi, Italy; c.settembre4@studenti.unisa.it (C.S.); dantonio.elvira@gmail.com (E.D.); 2 Rheumatology Unit, AOU San Giovanni di Dio e Ruggi d’Aragona, 84125 Salerno, Italy; moscatopaolo1@gmail.com (P.M.); loy.74@libero.it (G.L.)

**Keywords:** irritable bowel syndrome, functional dyspepsia, fibromyalgia, gastrointestinal diseases

## Abstract

The disorders of gut–brain interaction (DGBI) have been associated with Fibromyalgia (FM). However, there are no data about the relationship between FM and DGBI using Rome IV criteria. This study aimed to evaluate the prevalence of FM in patients with Irritable Bowel Syndrome (IBS) and/or Functional Dyspepsia (FD) and the prevalence of IBS and FD in FM patients using Rome IV criteria. DGBI patients and FM patients were recruited from two outpatient clinics devoted to DGBI and FM. All patients underwent a standardized gastrointestinal (GI) symptoms questionnaire. FM symptoms in DGBI patients were assessed through Fibromyalgia Rapid Screening Tool (FiRST) and Fibromyalgia Impact Questionnaire. Thereafter, the rheumatologists evaluated them. 49.0% of FM patients fulfilled the diagnostic criteria for IBS, 81.6% for FD with an overlap for both IBS/FD in 44.9%. IBS-C was the most prevalent IBS-subtype in DGBI patients, whereas IBS-M was the most prevalent in FM patients (*p* = 0.01). 45.3% of DGBI patients reported pathological FiRST scores. DGBI patients with FM showed the highest score at the standardized GI questionnaire followed by FM patients with DGBI and DGBI without FM. In conclusion DGBI are common in FM patients and vice versa. The presence of FD is extremely frequent in FM patients. A multidisciplinary approach should be routinely used for the management of these patients.

## 1. Introduction

The disorders of gut–brain interaction (DGBI) are a heterogeneous group of disorders characterized by gastrointestinal (GI) symptoms related to any combination of the following: motility disturbance, visceral hypersensitivity, altered mucosal and immune function, altered gut microbiota, and altered Central Nervous System (CNS) processing [1]. DGBI include several conditions such as irritable bowel syndrome (IBS), and functional dyspepsia (FD) [1]. Although DGBI share common physiological characteristics, they may differ for bodily location, duration of symptoms, and vary across individuals or within the same individual over time. Thus, the effective management of DGBI requires a biopsychosocial approach that addresses the variability and complexity of patients who have these disorders [2]. These conditions affect up to 30–40% of the general population in Western countries and decrease with age [3]. DGBI represent 12% of the workload in primary care and 30% of gastroenterological outpatient consultations [4]. The presence of DGBI is often associated with chronic pain syndromes such as Fibromyalgia (FM) and other functional syndromes (e.g., temporomandibular joint disorder, interstitial cystitis, chronic fatigue syndrome) [5,6].

FM is a central sensitization syndrome characterized by the altered perception, transmission, and processing of nociceptive stimuli. It is a very complex chronic condition characterized by musculoskeletal pain, but also by deep fatigue and numerous other symptoms affecting various organs and systems such as headache and facial pain, chest pain, stiffness, sensation of swelling, paraesthesia, skin changes, gastrointestinal or genitourinary disorders (irritable bladder syndrome), sleep disturbances, cognitive disturbances, anxiety and depression [7].

FM can be either primary, also known as idiopathic fibromyalgia, or secondary, that occurs in association with underlying disorders such as ankylosing spondylitis, trauma, or surgery. Previous studies demonstrated that primary and secondary FM are essentially equivalent regarding symptom burden [8,9].

The prevalence of FM in patients with IBS varies across the studies ranging from 28% up to 65% [10,11] depending on the different diagnostic criteria. In the same way, the prevalence of IBS in FM patients varied from 32% to 80% [12,13]. There is only one previous study addressing the prevalence of FD, according to Rome II criteria, in FM patients [14]. Furthermore, there are no studies evaluating either the prevalence of DGBI, diagnosed based on the recent Rome IV criteria, in the FM population or the prevalence of FM in the DGBI population.

Therefore, this study aimed to evaluate the prevalence of FM in patients with IBS and/or FD and, conversely, the prevalence of IBS and FD in FM patients according to the recent Rome IV criteria.

## 2. Materials and Methods

### 2.1. Population

Fifty-three DGBI patients and 49 FM patients were recruited from two outpatient clinics devoted to DGBI and FM, respectively, from a tertiary center, the University Hospital “San Giovanni di Dio e Ruggi d’Aragona” of Salerno.

Demographic characteristics (gender, age, and smoking habits), anthropometric data (weight, height, and BMI), and prevalence of comorbidities, i.e., hypertension, dyslipidemia, type 2 diabetes mellitus, and thyroid diseases, were collected at enrollment. Type of medications used was recorded.

FD and IBS were diagnosed by two experienced gastroenterologists in the field of DGBI according to Rome IV criteria [1] in all patients, together with the exclusion of any organic disease, with a complete physical examination, blood tests, and additional tests when indicated.

Four different patterns of IBS resulted from the predominant bowel symptom: (a) diarrhea predominant (IBS-D); (b) constipation predominant (IBS-C); (c) mixed IBS (IBS-M); and (d) undetermined IBS (IBS-U). The two considered FD subgroups were the Postprandial Distress Syndrome (PDS) and the Epigastric Pain Syndrome (EPS).

The characteristic symptoms of PDS were bothersome postprandial fullness or early satiation and those of EPS were unexplained epigastric pain or burning.

FM diagnosis was based on the American College of Rheumatology criteria [15]. All patients underwent the questionnaires described below.

### 2.2. Questionnaires

Standardized GI symptoms questionnaire. A previously published standardized questionnaire dealing with the presence, the frequency from 0 to 3 (0 = absent, 1 = ≤2 d/wk; 2 = 3–5 d/wk; and 3 = ≥6 d/wk), and the intensity from 0 to 3 (0 = absent; 1 = not very bothersome, not interfering with daily activities; 2 = bothersome, but not interfering with daily activities; and 3 = interfering with daily activities), of a number of upper and lower GI symptoms, was used in all patients [16,17]. For each symptom, a frequency-intensity score from 0 up to a maximum of 6 was obtained. Stool consistency was recorded as numerical value using the Bristol Stool Form Scale (BSFS). Daily measurement of the number of bowel movements was summarized weekly.

Fibromyalgia Rapid Screening Tool (FiRST) questionnaire was used to screen FM in DGBI patients (Table A1) [18]. FiRST is a quick and effective test developed and validated by the French Society of Rheumatology, used to detect FM in <3 min with a sensitivity of 90.5% and specificity of 85.7% [19]. It consists of six items related to several FM dimensions: widespread pain (item 1), fatigue (item 2), pain characteristics (item 3), non-painful abnormal sensations (item 4), functional somatic symptoms (item 5), sleep and cognitive problems (item 6). Each affirmative answer was associated to a score of 1 point, whereas 0 points were calculated for negative answers. The maximum score obtainable with the test was 6 points, but a cut-off of 5 is associated with a correct identification of patients.

Fibromyalgia Impact Questionnaire (FIQ). The Italian validated version of FIQ was used to assess the overall impact of FM on the different dimensions of the patient’s life [20].

It consists of 10 items that measures physical functioning, work status, depression, anxiety, sleep, pain, stiffness, fatigue, and well-being, with a maximum score of 10 for item and a maximum overall score of 100, with 100 indicating maximum FM impact [21].

In addition to the FiRST and FIQ questionnaires, patients with DGBI were evaluated by the rheumatologist to confirm the diagnosis of FM.

### 2.3. Statistic Analysis

Results are expressed as frequencies, median and interquartile range (IQR), unless otherwise indicated. When appropriate, a χ2 test to compare categorical data and analysis of variance (ANOVA) to compare continuous variables were used. Significance was expressed at *p* < 0.05 level. SPSS for Windows (release 15.0; SPSS Inc. Chicago, IL, USA) was used for statistical analysis

## 3. Results

Demographic characteristics, anthropometric data, and prevalence of comorbidities in DGBI and FM patients were shown in Table 1.

None of DGBI patients used antidepressants and analgesics. Patients with primary FM were newly diagnosed and did not use antidepressant and analgesics. Patients with secondary FM were on appropriate treatment for their underlying disorders: ada-limumab and golimumab (anti TNF-alfa); abatacept (CTLA4-Ig fusion protein); Tofa-citinib (Janus kinase inhibitor); and Ixekizumab (IL-17A antagonist).

Among DGBI patients, 37/53 (69.8%) fulfilled the Rome IV criteria for the diagnosis of IBS, 41 (77.4%) for FD, and 27 (50.9%) for both IBS and FD. Fifteen patients (40.5%) fulfilled the diagnostic criteria for IBS-C, 11 (29.7%) for IBS-M, and 11 (29.7%) for IBS-D.

Twenty-four/53 patients (45.3%) DGBI patients reported a FiRST score ≥ 5, and a mean FIQ score of 51.48 ± 16.55. Only 9/24 (37.5%) patients agreed to undergo a rheumatological examination. The diagnosis of primary FM was confirmed in all 9 patients.

Among FM patients, 19/49 (38.7%) patients were diagnosed as primary FM, while 30 (61.3%) patients as secondary FM associated with clinical conditions such as ankylosing spondylitis (6.7%), monoclonal gammopathy (3.3%), oligoarthritis (3.3%), or undifferentiated connectivity (13.3%) in clinical and laboratory remission through the use of appropriate treatment.

Twenty-four/49 (49.0%) FM patients fulfilled the Rome IV diagnostic criteria for IBS, 40/49 (81.6%) for FD with an overlap for both IBS/FD in 22/49 (44.9%) patients. There are no significant differences in the prevalence of IBS and FD in patients with primary and secondary FM (*p* > 0.05).

**Figure 1:**
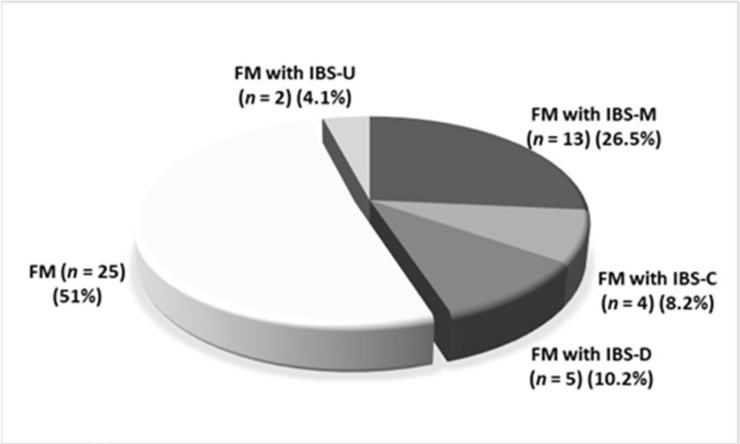
Prevalence of subtypes of IBS among FM patients. FM: fibromyalgia; IBS: irritable bowel syndrome; IBS-U: undetermined IBS; IBS-M: mixed IBS; IBS-C: constipation predominant IBS; IBS-D: diarrhea predominant IBS.

Figure 1 showed the prevalence of IBS and its subtypes among FM patients (*n* = 49).

**Figure 2:**
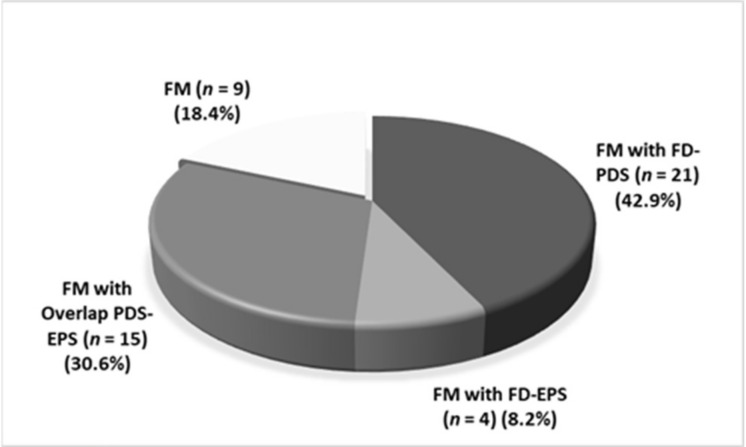
Prevalence of subtypes of FD among FM patients. FM: fibromyalgia; FD: functional dyspepsia; PDS: postprandial distress syndrome; EPS: epigastric pain syndrome.

Figure 2 showed the prevalence of FD and its subtypes among FM patients (*n* = 49).

There was a statistical difference among IBS subtypes between DGBI and FM patients; In fact, IBS-C was the more prevalent IBS subtype in DGBI patients, whereas IBS-M was the most prevalent in FM patients (*p* = 0.01). Although there was a difference in FD subtypes between DGBI (PDS 39.6%, EPS 20.8%, 17% overlap PDS and EPS) and FM patients (Figure 2), it did not reach the statistical significance. Interestingly, there was a low prevalence of EPS in FM patients.

All recruited patients underwent the standardized GI symptoms questionnaire to evaluate both upper and lower GI symptoms.

Table 2 showed the frequency intensity scores of the studied GI symptoms in FM patients with DGBI, FM patients without DGBI, DGBI patients with FM, and DGBI patients without FM. Data were expressed as median (IQR).

A significant difference in the frequency-intensity scores of upper abdominal symptoms such as epigastric fullness, pain and burning, upper abdominal bloating and distension was found among all groups with graduated scores. DGBI patients with FM showed the highest score followed by FM patients with DGBI and DGBI without FM. A significant difference was found among the lower abdominal symptoms only in abdominal pain and DGBI without FM had the highest score. Moreover, the frequency-intensity scores of the GI symptoms did not significantly differ between primary and secondary FM patients (*p* > 0.05).

## 4. Discussion

IBS and FD are the most common DGBI with a prevalence ranging from 3.9% to 4.2% and from 7.0% to 7.4%, respectively, according to the Rome IV criteria [12]. It is well known that FM, an extraintestinal chronic pain disorder characterized by widespread pain, is frequently associated with IBS with higher severity of illness [12,22].

Both FM and DGBIs shared common clinical characteristics: they are prevalent in women and significantly impact the quality of life, contributing to high levels of work absenteeism, seeking health assistance and a significant financial burden. It is thought that both DGBIs and FM recognize as the main common pathophysiological mechanism the abnormal and intense amplification of pain by the CNS (central sensitization) associated with an abnormal perception of stimuli from the periphery (skin, muscles, tendons, internal organs) [19]. Moreover, Sperber et al. [11] reported that FM with IBS have worse symptoms of fatigue and morning pain, compared to those without IBS. This is consistent with the other reports linking the worsening of GI symptoms during periods of exacerbations of FM [20]. However, beyond IBS, the relationship between FM and other DGBI remains largely underexplored. This study demonstrated in FM patients a high prevalence of the most common DGBI such as IBS and FD accordingly to the latest Rome IV criteria and a high prevalence of FM both in IBS and FD patients. Our novel results were that, a part from an high frequency of IBS, also FD was extremely prevalent, up to 80% in FM patients with a graduated increase of the intensity-frequency of several upper GI symptoms when FM and DGBI coexisted.

A recent systematic review in 2020 [23] compared the data of the fourteen most significant studies (since 1978) that report the prevalence of DGBI in subjects with FM, predominantly female and with an age between 29 and 56 years. It emerged that half of FM patients have at least one DGBI with a heavy weighting of IBS prevalence data.

Although the prevalence of DGBI varied widely due to different diagnostic criteria and population, the pooled data demonstrated an overall prevalence of DGBI of 50.8% and 46.2%, for IBS in people with FM that was closely aligned with the 45.3% found in our study.

Among these studies, only one [13] described FD, but with a lower prevalence, about 21%, of FD, accordingly to Rome II criteria and, to our knowledge, no studies evaluated FD subtypes.

Another finding of our study was that IBS-M was the significantly most common subtype in our FM patients. This result is in key with other studies [23].

Previous study already demonstrated using an electronic barostat that IBS have a graduated visceral sensitivity based on the severity of illness; however, the presence of FMS that co-exists only with a more severe IBS attenuated visceral sensitivity [24,25]. Our results supported these findings, showing that the intensity-frequency of abdominal pain was higher in DGBI patients without FM.

However, the pathophysiological mechanisms underlying these two pathologies remains, to date, still underexplored. Among the proposed mechanisms, there are changes in the nociceptive system that result in an increased pain (hyperalgesia and allodynia) or modifications in immune system that could lead to an increased inflammation or other factors such as sleep disturbances, fatigue, anxiety, and headache that can influence the pain in FM patients [26].

Another suggested pathophysiologic mechanism, in fact, is the alteration in the brain–gut axis occurring via Small Intestinal Bacterial Overgrowth (SIBO), which is frequently described in FM patients, or subclinical infections such as giardiasis [26,27].

Therefore, the treatment of the fibromyalgia patient should be focused both on these peripheral and central components, in addition to the improvement of the associated conditions, such as the improvement of quality of sleep or of anxiety levels [26,27].

This study has several limitations. Firstly, the low number of subjects still poses uncertainty about the extent of observed phenomena. Secondly, this study represents tertiary care patients and it might impact the generalizability of our results. Another limitation is the low number of DGBI patients with pathological FiRST and FIQ scores, who agreed to undergo a rheumatological examination to confirm the FM diagnosis. However, FiRST is a validated test with a very high sensitivity and specificity. Lastly, we did not specifically assess psychiatric diagnoses in all patients, although it is well known the association between mental disorders and both DGBI and FM.

## 5. Conclusions

In conclusions, we confirmed the bidirectional relationship between DGBI and FM, with different prevalence rates compared to previous studies and new scenarios for the future.

Therefore, researches exploring the association between FM and the full range of DGBIs, are warranted.

The result of this experimental study will later allow us to have a multiplinary strategy for the early diagnosis and treatment of patients with these comorbidities, reducing the costs associated with these diseases and increasing the quality of life of these patients.

## Figures and Tables

**Table 1:** Demographic characteristics, anthropometric data, and prevalence of comorbidities in DGBI and FM patients. Data were expressed as frequencies or mean ± standard deviation.

	DBGI Patients	FM Patients	*p*
Patients (N°)	53	49	
Sex (*n*, %) - M - F	16 (30.2%) 37 (69.8%)	4 (8.2%) 45 (91.8%)	0.005
Age (Years)	45.7 ± 16.3	56.3 ± 13.0	0.001
Weight (Kg)	65.8 ± 14.7	71.6 ± 15.2	0.060
BMI (kg/m^2^)	23.9 ± 4.5	28.2 ± 5.6	0.001
Smoking habits (yes, %)	12 (22.6%)	16 (38.1%)	0.1
Comorbidity (n, %) - Hypertension - Type 2 Diabetes mellitus - Dyslipidemia - Thyroid disease	7 (13.2%) 0% 6 (11.3%) 7 (13.2%)	7 (14.3%) 6 (12.2%) 9 (18.4%) 5 (10.2%)	0.600 0.004 0.300 0.800

**Table 2:** Frequency intensity scores of GI symptoms.

Symptoms	FM Pts without DGBI *n = 7*	FM Pts with DGBI *n = 42*	DGBI Pts with FM *n = 24*	DGBI Pts without FM *n = 29*	*p*
Epigastric fullness	0.0 (0.0–0.0)	3.5 (0.0–5.0)	4.0 (0.0–5.0)	3.0 (0.0–5.0)	0.024
Early satiety	0.0 (0.0–0.0)	0.0 (0.0–4.0)	0.0 (0.0–4.8)	0.0 (0.0–0.0)	0.132
Epigastric pain	0.0 (0.0–0.0)	0.0 (0.0–2.0)	2.0 (0.0–4.0)	0.0 (0.0–2.0)	0.049
Epigastric burning	0.0 (0.0–0.0)	0.0 (0.0–2.0)	1.0 (0.0–4.0)	0.0 (0.0–1.0)	0.019
Nausea	0.0 (0.0–0.0)	0.0 (0.0–2.0)	0.0 (0.0–3.5)	0.0 (0.0–2.0)	0.568
Upper abdominal bloating	0.0 (0.0–0.0)	4.0 (2.8–5.0)	4.5 (2.0–3.0)	3.0 (0.0–5.0)	0.001
Upper abdominal distension	0.0 (0.0–0.0)	4.0 (2.8–5.0)	4.5 (2.0–5.0)	3.0 (0.0–5.0)	0.002
Abdominal pain	0.0 (0.0–0.0)	2.0 (0.0–4.0)	2.5 (0.0–4.0)	3.0 (0.0–4.0)	0.036
Number of weekly evacuations	7.0 (3.0–7.0)	7.0 (7.0–14.0)	7.0 (3.3–7.0)	7.0 (4.5–14.0)	0.091
Bristol stool scale	2.0 (2.0–4.0)	4.0 (2.8–5.0)	3.0 (2.0–4.0)	4.0 (2.0–5.0)	0.420
Sensation of incomplete evacuation	2.0 (0.0–5.0)	2.5 (0.0–4.3)	4.0 (0.0–5.0)	2.0 (0.0–4.0)	0.527
Straining during defecation	2.0 (2.0–4.0)	2.0 (0.0–3.0)	3.0 (0.0–4.0)	0.0 (0.0–4.0)	0.358
Sensation of anorectal obstruction/blockage	0.0 (0.0–2.0)	0.0 (0.0–2.0)	0.0 (0.0–3.8)	0.0 (0.0–0.0)	0.372
Lower abdominal bloating	0.0 (0.0–0.0)	3.0 (0.0–4.0)	3.0 (0.0–4.0)	3.0 (0.0–4.0)	0.080
Lower abdominal distension	0.0 (0.0–0.0)	3.0 (0.0–4.0)	3.5 (0.0–4.0)	0.0 (0.0–4.0)	0.075
Tenesmus	0.0 (0.0–2.0)	1.0 (0.0–2.0)	0.0 (0.0–3.8)	0.0 (0.0–2.0)	0.400

FM: fibromyalgia; Pts: patients; DGBI: disorder of gut-brain interaction

## Data Availability

No additional data are available.

## Appendix A

**Table A1:** The Fibromyalgia Rapid Screening Tool (FiRST).

	Yes	No
1—I have pain all over my body		
2—My pain is accompanied by a continuous and very unpleasant general fatigue		
3—My pain feels like burns, electric shocks or cramps		
4—My pain is accompanied by other unusual sensations throughout my body, such as pins and needles, tingling or numbness		
5—My pain is accompanied by other health problems such as digestive problems, urinary problems, headaches or restless legs		
6—My pain has a significant impact on my life, particularly on my sleep and my ability to concentrate, making me feel slower generally		
